# Ready, Set, Screen: Evaluating Community Readiness for Lung Cancer Screening Among Non-Hispanic Black Populations in Florida: Brief Report

**DOI:** 10.1016/j.jtocrr.2026.100971

**Published:** 2026-02-11

**Authors:** Shanada Monestime, Annesha White, Rachelle Theodore, Luis E. Raez, Courtney A. Granville, Donna Harris, Jennifer C. King

**Affiliations:** a GO2 for Lung Cancer, Washington, DC; b University of North Texas Health Science Center, Fort Worth, Texas; c CLT Business Solutions, Homestead, Florida; d Memorial Cancer Institute, Hollywood, Florida; e Villages at NorthStar Community Development Corporation, Detroit, Michigan

**Keywords:** Lung cancer screening, Community engaged research, Social determinants of health, Community readiness, Blacks, Health care access

## Abstract

**Introduction:**

Lung cancer is the leading cause of cancer death in the United States, with non-Hispanic Black populations facing disproportionately high mortality and low lung cancer screening (LCS) rates. Despite national efforts to expand screening, limited evidence on community readiness may constrain implementation. This study assessed community readiness to implement LCS in Black communities in Broward County, Florida.

**Methods:**

We used the community readiness model, which scores the following five dimensions: knowledge of efforts, leadership, community climate, knowledge of the issue, and resources, on a nine-point scale from 1 (“no awareness”) to 9 (“high community ownership”). From June to August 2023, we conducted semi-structured interviews with 19 purposively sampled leaders across community, health care, academic, and government sectors. Two trained coders independently scored transcripts; consensus scores were averaged to determine readiness.

**Results:**

The mean overall readiness score was 1.92, corresponding to the “no awareness” stage. Community knowledge of LCS efforts was minimal (1.24), with participants noting limited outreach despite available screening services. Leadership engagement (1.92) was low, with LCS found as a lower priority amid other health demands. Knowledge of lung cancer (1.82) was limited, shaped by misinformation and lack of awareness. Community climate (1.61) reflected barriers such as cost, lack of insurance, and caregiving burdens. Resources scored higher (3.03), but existing assets remained uncoordinated and underutilized.

**Conclusion:**

Black communities in Broward County demonstrated low readiness for LCS, driven less by hesitancy and more by invisibility. Building readiness will require targeted awareness building, leadership engagement, and coordination of existing resources before pursuing uptake-focused implementation.

## Introduction

Lung cancer remains the leading cause of cancer-related deaths in the United States, claiming more lives annually than breast and colorectal cancers combined.^1^ Despite the availability of early detection through low-dose computed tomography (LDCT), screening rates remain alarmingly low.^2^ In Florida, only 18.0% of individuals at high risk undergo screening, placing the state 29th among U.S. states and highlighting persistent gaps in screening uptake.^3^ Although state-specific data on lung cancer screening (LCS) rates within Black communities in Florida are limited, national trends consistently reveal that Black Americans face disproportionately higher lung cancer incidence and mortality rates compared with other racial or ethnic minority groups.^4^ These disparities highlight the need to understand factors contributing to low uptake in Black communities.

Although several studies have implemented interventions to improve LDCT uptake, most focused on enhancing knowledge, awareness, and access through patient education, streamlined clinic workflows, and community partnerships.^5^, ^6^, ^7^, ^8^ Despite these efforts, limited research has evaluated whether communities are prepared to implement LCS interventions or assessed their readiness to adopt such strategies. Readiness assessments have been widely used in public health to ensure interventions align with a community’s needs, resources, and capacity for change. Studies in other areas, such as smoking-free polices, cancer prevention, and palliative care,^9^, ^10^, ^11^ have demonstrated the importance of understanding a community’s level of preparedness before implementing interventions, as strategies that do not align with readiness levels may be less effective or unsustainable. However, to our knowledge, no studies have assessed community readiness for LCS within Black populations. This study represents the first phase of READY Lung (Readiness of Elevating African American/Black Communities to Develop an Initiative to Yield Change in Lung Cancer Disparities), a multi-phase, community-engaged program focused on assessing readiness for LCS and informing dissemination strategies in Black communities. Understanding the stage of readiness allows for the development of targeted strategies that align with community needs and optimize intervention effectiveness.

## Methods

This mixed-method study used the community readiness model (CRM) to assess the readiness of Black communities to engage in LCS efforts.^12^ Developed by the Tri-Ethnic Center for Prevention Research, the CRM evaluates community readiness across the following five dimensions: (1) community knowledge of efforts, (2) leadership, (3) community climate, (4) community knowledge of the issue, and (5) resources. Each dimension is scored on a scale from 1 (no awareness) to 9 (high community ownership). Table 1 provides an overview of the CRM dimensions and scoring criteria. Broward County, Florida, was selected because of persistent cancer disparities and structural barriers affecting Black residents, including limited access to care, socioeconomic challenges, and disproportionately high cancer mortality rates.^13^ Approximately 28% of residents identify as Black, exceeding the national average and highlighting the relevance of this setting. The county has seven facilities capable of providing LCS, which is fully covered for eligible insured individuals, with reduced-cost self-pay options available at some sites.

**Table 1:** Nine Stages of Community Readiness With Corresponding Scores

Stage (Readiness Score)	Description	Goal
No awareness (1–1.99)	Lung cancer screening is not generally recognized by the community or leaders as a problem (or it may truly not be an issue).	Raise awareness of the issue.
Denial/resistance (2–2.99)	At least some community members recognize that lung cancer screening is a concern, but there is little recognition that it might be needed locally.	Raise awareness of the problem and how it affects the community.
Vague awareness (3–3.99)	Most feel that there is local concern, but there is no immediate motivation to do anything about lung cancer screening.	Raise awareness that the community can help address the problem.
Preplanning (4–4.99)	There is clear recognition that something must be done about lung cancer screening, and there may even be a group addressing it. However, efforts are not focused or detailed.	Raise awareness with concrete plans to address the problem.
Preparation (5–5.99)	Active leaders begin planning in earnest. The community offers modest support of lung cancer screening efforts.	Gather existing information to develop a concrete plan of action.
Initiation (6–6.99)	Enough information is available to justify lung cancer screening efforts. Activities are underway.	Provide information that is tailored for the specific community.
Stabilization (7–7.99)	Lung cancer screening activities are supported by administrators or community decision makers. Staff are trained and experienced.	Stabilize efforts to address the problem.
Confirmation/expansion (8–8.99)	Lung cancer screening efforts are in place. Community members feel comfortable using services, and they support expansions. Local data are regularly obtained.	Expand and enhance services.
Community ownership (9–9.99)	Detailed and sophisticated knowledge exists about lung cancer screening prevalence and consequences. Effective evaluation guides new directions. The model is applied to other issues.	Maintain programs and continue to expand.

Between June and August 2023, 19 semi-structured interviews were conducted virtually with community leaders selected across multiple sectors using the local public health system partners (“Jelly Bean”) framework, including health care, community- and faith-based, advocacy, and government organizations.^14^ Although the CRM recommends six to 12 interviews, we conducted additional interviews using purposive sampling to ensure representation across all Jelly Bean sectors relevant to LCS. Organizations nominated a representative in response to e-mail invitations who identified as Black or worked closely with Black communities, spoke English, and were involved in health-related community engagement or implementation activities. Some participants both lived and worked in the communities they served, providing community-embedded perspectives alongside organizational insight. Participants received a $100 honorarium. The study was deemed exempt by WCG IRB.

Interviews lasted approximately 90 minutes and were conducted by a trained moderator and facilitator. All participants provided written consent before data collection. Interview sessions were recorded and transcribed verbatim. Two trained coders independently reviewed each transcript and scored responses using CRM criteria. Discrepancies were resolved by consensus. Average scores were calculated for each dimension and then aggregated to determine the overall community readiness level. Full scoring procedures are detailed in the CRM Handbook.^12^

## Results

### Participant Characteristics and Community Readiness Score

**Figure 1:**
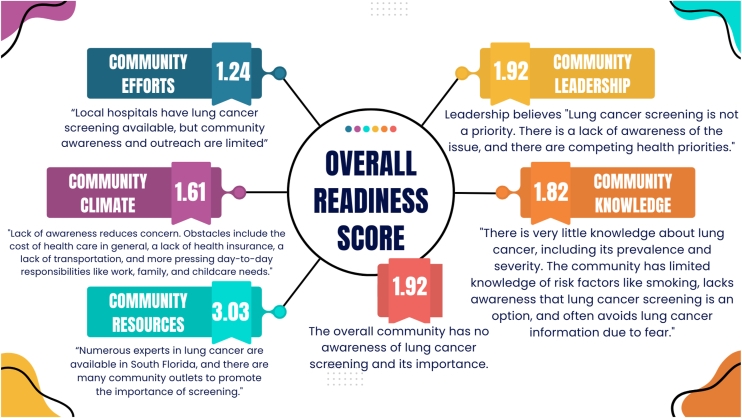
Community readiness scores across five domains related to lung cancer screening. Illustrates domain-specific scores and quotes from interviews assessing awareness, leadership, climate, knowledge, and resources within a non-Hispanic Black community in Broward County, Florida.

A total of 19 community leaders participated in the interviews. Most were between 35 and 49 years old (47.3%), female (63.2%), and identified as Black (52.6%). Most represented community organizations (47.4%) and reported a moderate level of engagement with Black communities (57.9%). On average, participants had worked in Broward County for 11.5 years (Table 2). The overall community readiness score was 1.92, which corresponds to the “no awareness” stage—the lowest level on the CRM scale. This suggests that LCS is not viewed as a public health priority, with limited recognition of the issue or related efforts. Dimension scores and illustrative quotes appear in Table 3 and Figure 1.

**Table 2:** Characteristics of the Community Members

Baseline Characteristics N = 19	
Age, n (%)	
50–80	8 (42.1)
35–49	9 (47.3)
18–34	2 (10.5)
Race, n (%)	
Black/African American	10 (52.6)
White	7 (36.8)
Other	2 (10.5)
Ethnicity, n (%)	
Hispanic	7 (36.8)
Non-Hispanic	12 (63.2)
Sex, n (%)	
Male	7 (36.8)
Female	12 (63.2)
Extent of effort in engaging with Black/African American communities, n (%)	
Moderate	11 (57.9)
A lot	8 (42.1)
Organization type	
Community, faith-based, and neighborhood organizations	9 (47.4)
Health care, clinical, academic institutions, and private sector/employers	6 (31.6)
Public health, government, and policy	2 (10.5)
Advocacy and nonprofit health organizations	2 (10.5)
Years working in Broward County, mean (SD)	11.59 (±10.20)

**Table 3:** Community Readiness Score for Lung Cancer Screening in the Black Community

Dimension	Interview Participants																				Stage of Readiness
	1	2	3	4	5	6	7	8	9	10	11	12	13	14	15	16	17	18	19	Avg.	
Knowledge of efforts	1.5	1	1	2	1	1	1	1	1	2	1	1	1	1	1	1	1	1	3	1.24	No awareness
Leadership	1	1	1	3.5	1	2	1	2	3	1	1	1	4	5	1	1	1	1	2	1.92	No awareness
Community climate	1.5	1	1	1	3	1	1	2	3	2	1	4	1	1	1	1	1	2	1	1.61	No awareness
Knowledge of issue	3	2.5	2	1.5	1	2	2	2	2	2	2	2	2	2	2	1	2	1.5	2	1.82	No awareness
Resources	3	2.5	3	3	3	2	3	4	3	4	3	2	3	2	3	2	6	7	2	3.03	Vague awareness
Overall community readiness score																				1.92	No awareness

### Knowledge of Efforts and Leadership Engagement

Community knowledge of efforts to promote LCS was notably limited, with a mean score of 1.24—among the lowest across all dimensions. Most participants were unaware of any organized LCS programs in their area, and those who had encountered such efforts described them as sporadic and poorly attended. In contrast, smoking cessation efforts were more widely recognized, with participants reporting greater exposure to tobacco-related messaging than to cancer prevention. Leadership support mirrored these patterns of inattention. With an average score of 1.92, community leaders and decision-makers were perceived as minimally involved in addressing LCS. This lack of engagement was often attributed to competing priorities, including substance use, economic instability, and other pressing health and social challenges. Participants noted that LCS rarely surfaces on the policy agenda or in funding discussions, highlighting a need for advocacy to elevate the issue within leadership circles.

### Knowledge of the Issue and Community Climate

Community knowledge of the issue—that is, general awareness and understanding of lung cancer and screening guidelines—received an average score of 1.82. Although most respondents were aware that lung cancer exists, they often associated it narrowly with smoking and demonstrated limited understanding of additional risk factors or of LDCT as a screening tool. Misinformation, fear, and stigma surrounding cancer were also cited as barriers to seeking accurate information or care. In contrast to breast or cervical cancer, which benefit from robust public education campaigns, lung cancer remains underrepresented in community discourse. The community climate dimension scored 1.61, reflecting limited public concern or motivation regarding LCS. Respondents described how day-to-day stressors—such as financial strain, work, and caregiving responsibilities and limited access to care—often overshadow preventive health behaviors. When LCS is not publicized or integrated into routine health care, it becomes even less likely to be prioritized.

### Community Resources

The only dimension that deviated slightly from the “no awareness” range was resources, which received an average score of 3.03—classified as “vague awareness.” Several participants acknowledged that the community possesses valuable assets that could support future LCS initiatives, including physical spaces for outreach, local medical professionals with expertise in lung cancer, and established community-based organizations. However, these resources are not currently aligned or mobilized around LCS. The underutilization of these assets, combined with a lack of dedicated funding or cross-sector coordination, limits the community’s ability to implement sustainable LCS efforts.

## Discussion

This study identified a critical gap: LCS readiness is driven less by hesitancy and more by invisibility, exhibited by limited awareness across all five CRM dimensions—knowledge of efforts, leadership, climate, issue awareness, and resources. This lack of awareness is concerning, given well-documented disparities in lung cancer outcomes, where Black individuals are more likely to be diagnosed at later stages and experience higher mortality rates compared with other racial or ethnic groups.^4^ Although national guidelines have expanded LCS eligibility to increase access among high-risk populations, awareness and implementation continue to lag, particularly in racially diverse communities.^15^ Recent analyses suggest that even expanded criteria may perpetuate disparities in screening access and benefit, especially among Black individuals.^16^ Although existing literature emphasizes health beliefs and psychosocial factors—such as stigma, access barriers, and fear of false positives—influence the decision to opt out of LCS, other studies, including ours, suggest that some individuals never reach that point due to a more fundamental barrier: lack of awareness that screening exists or is relevant.^8^^,^^17^, ^18^, ^19^

When organized LCS efforts are unknown to community leaders and absent from public health or policy agendas, low uptake is unsurprising. Respondents reported greater familiarity with smoking cessation programs, suggesting that although tobacco messaging has reached these communities, cancer screening has not. This imbalance likely reflects Florida’s sustained investment in tobacco control, supported by an annual budget exceeding $80 million as of 2025, dedicated to community-based prevention, policy initiatives, and smoking cessation services, whereas LCS lacks a comparable statewide framework or level of investment. In addition, the community climate score (1.61) reflected a broader sense of inertia; residents facing economic pressures, caregiving burdens, and competing health priorities often deprioritize early detection services, including cancer screening. These findings align with literature on the social determinants of health, which emphasize structural barriers that disproportionately affect marginalized populations.^20^ Our findings suggest that CRM scoring can serve as a diagnostic tool to guide when and how to engage communities in public health campaigns. This approach supports more strategic allocation of resources, moving beyond universal outreach toward targeted, stage-specific intervention. At a readiness score of 1.92, consistent with the “no awareness” stage of the CRM, recommended strategies emphasize early awareness building rather than behavior change. This includes engaging trusted community stakeholders to determine how information about LCS should be delivered in ways that are relatable, culturally acceptable, and trusted by the community, before introducing efforts designed to increase screening participation. These efforts are particularly important given that LCS is not currently included in Centers for Medicare and Medicaid Services Health Effectiveness Data and Information Set quality measures, which may limit prioritization and reinforcement of LCS within primary care settings and contribute to persistently low uptake nationwide.

Although this study provides valuable insights into community readiness for LCS, it has some limitations. The interview guide was structured around the five CRM dimensions, which limited insights to those specific areas. As a result, broader influences on screening, such as systemic racism, historical mistrust, and competing health priorities, may not have been fully captured. Nonetheless, the study offers key strengths; it applies a validated readiness framework to LCS and incorporates diverse, cross-sector community perspectives. Future research should test context-specific strategies to improve readiness and support equitable implementation in underserved communities.

In conclusion, this study offers novel insights into LCS readiness in Black communities using a structured, community-based assessment. Readiness was low, reflecting limited awareness of LCS despite the presence of screening infrastructure, suggesting that low uptake is driven less by hesitancy and more by invisibility. At a “no awareness” readiness stage, efforts should engage trusted community members to guide how and where foundational LCS information is shared before pursuing strategies to increase screening rates.

## CRediT Authorship Contribution Statement

**Shanada Monestime:** Conceptualization, Data curation, Formal analysis, Funding acquisition, Methodology, Project administration, Visualization, Writing – original draft, Writing – review & editing.

**Annesha White:** Conceptualization, Methodology, Supervision, Writing – review & editing.

**Rachelle Theodore:** Data curation, Formal analysis, Writing – review & editing.

**Luis E. Raez:** Conceptualization, Data curation, Writing – review & editing.

**Courtney A. Granville:** Formal analysis, Supervision, Validation, Writing – review & editing.

**Donna Harris:** Conceptualization, Methodology, Writing – review & editing.

**Jennifer C. King:** Conceptualization, Methodology, Supervision, Writing – review & editing.

## Disclosure

The authors declare no conflict of interest.

## References

[1] American Cancer Society (2025). https://www.cancer.org/content/dam/cancer-org/research/cancer-facts-and-statistics/annual-cancer-facts-and-figures/2025/2025-cancer-facts-and-figures-acs.pdf.

[2] Poon C., Wilsdon T., Sarwar I., Roediger A., Yuan M. (2023). Why is the screening rate in lung cancer still low? A seven-country analysis of the factors affecting adoption. Front Public Health.

[3] American Lung Association State of lung cancer - key findings. https://www.lung.org/research/state-of-lung-cancer/key-findings.

[4] Ryan B.M. (2018). Lung cancer health disparities. Carcinogenesis.

[5] Nam J., Krishnan G., Shofer S., Navuluri N. (2023). Interventions to improve lung cancer screening among racially and ethnically minoritized groups: a scoping review. Lung Cancer.

[6] Lau Y.K., Bhattarai H., Caverly T.J. (2021). Lung cancer screening knowledge, perceptions, and decision making among African Americans in Detroit, Michigan. Am J Prev Med.

[7] Carter-Bawa L., Ostroff J.S., Erwin D.O. (2025). A community-based approach to address lung cancer screening disparities in the black community using the Witness Project® framework: development and pilot trial. BMC Public Health.

[8] Gudina A.T., Kamen C., Mattick L.J. (2024). Knowledge and beliefs about lung cancer screening among Black individuals at high risk: a qualitative approach. Transl Lung Cancer Res.

[9] Wainwright J.E., Cook E.J., Ali N., Wilkinson E., Randhawa G. (2024). Community readiness to address disparities in access to cancer, palliative and end-of-life care for ethnic minorities. BMC Public Health.

[10] Arambula Solomon T.G., Jones D., Laurila K. (2023). Using the community readiness model to assess American Indian communities readiness to address cancer prevention and control programs. J Cancer Educ.

[11] York N.L., Hahn E.J. (2007). The Community Readiness Model: evaluating local smoke-free policy development. Policy Polit Nurs Pract.

[12] Oetting E.R., BAP, Edwards R.W., Thurman P.J., Kelly K.J., Beauvais F. (2014).

[13] Perez J.M. State of Black Broward. https://https://tec.colostate.edu/wp-content/uploads/2018/04/CR_Handbook_8-3-15.pdff.

[14] National Association of Local Boards of Health National public health performance standards local implementation guide. version 3.0. https://www.naccho.org/uploads/downloadable-resources/Programs/Public-Health-Infrastructure/Governance-Handbook-FINAL-4-22-13.pdf.

[15] Zeliadt S.B., Hoffman R.M., Birkby G. (2018). Challenges implementing lung cancer screening in federally qualified health centers. Am J Prev Med.

[16] Young C.D., Katki H.A., Cheung L.C. (2025). Individual- and group-level disparities between racial and ethnic groups in lung cancer screening eligibility criteria. JAMA Netw Open.

[17] Schiffelbein J.E., Carluzzo K.L., Hasson R.M., Alford-Teaster J.A., Imset I., Onega T. (2020). Barriers, facilitators, and suggested interventions for lung cancer screening among a rural screening-eligible population. J Prim Care Community Health.

[18] Carter-Harris L., Brandzel S., Wernli K.J., Roth J.A., Buist D.S.M. (2017). A qualitative study exploring why individuals opt out of lung cancer screening. Fam Pract.

[19] Cavers D., Nelson M., Rostron J. (2022). Understanding patient barriers and facilitators to uptake of lung screening using low dose computed tomography: a mixed methods scoping review of the current literature. Respir Res.

[20] Elmohr M.M., Javed Z., Dubey P. (2024). Social determinants of health framework to identify and reduce barriers to imaging in marginalized communities. Radiology.

